# Impact on quality of life in teachers after educational actions for prevention of voice disorders: a longitudinal study

**DOI:** 10.1186/1477-7525-11-28

**Published:** 2013-02-27

**Authors:** Raquel Aparecida Pizolato, Maria Inês Beltrati Cornacchioni Rehder, Marcelo de Castro Meneghim, Glaucia Maria Bovi Ambrosano, Fábio Luiz Mialhe, Antonio Carlos Pereira

**Affiliations:** 1 Departament of Community Dentistry, Division of Health Education and Health Promotion, Piracicaba Dental School, University of Campinas- UNICAMP, PO. BOX 52, 13414-903, Piracicaba, SP, Brazil; 2 CEFAC- Graduate Center for Health and Education, São Paulo, SP, Brazil

**Keywords:** Occupational health, Teachers, Voice training, Quality of life, Questionnaire study

## Abstract

**Background:**

Voice problems are more common in teachers due to intensive voice use during routine at work. There is evidence that occupational disphonia prevention programs are important in improving the quality voice and consequently the quality of subjects’ lives.

**Aim:**

To investigate the impact of educational voice interventions for teachers on quality of life and voice.

**Methods:**

A longitudinal interventional study involving 70 teachers randomly selected from 11 public schools, 30 to receive educational intervention with vocal training exercises and vocal hygiene habits (experimental group) and 40 to receive guidance on vocal hygiene habits (control group control). Before the process of educational activities, the Voice-Related Quality of Life instrument (V-RQOL) was applied, and 3 months after conclusion of the activities, the subjects were interviewed again, using the same instrument. For data analysis, Prox MIXED were applied, with a level of significance α < 0.05. Results: Teachers showed significantly higher domain and overall V-RQOL scores after preventive intervention, in both control and experimental groups. Nevertheless, there was no statistical difference in scores between the groups.

**Conclusion:**

Educational actions for vocal health had a positive impact on the quality of life of the participants, and the incorporation of permanent educational actions at institutional level is suggested.

## Background

Voice problems in professionals who use their voice as an instrument for work, may directly affect the quality of the individual’s voice, interfering in social, emotional and physical aspects related to day-to-day life [1].

Studies with reference to vocal health and its impact on teachers’ quality of life have been of interest to researchers during the last decade, because among other professions, they are considered those who present greater risk for developing voice disturbances [1-3]. Symptoms such as hoarseness, vocal breaks, vocal fatigue, burning in the throat, and temporary aphonia are frequent manifestations in the health of these professionals [4], and these problems may interfere in the performance of their work and social relationships, causing frustration and low self-esteem [2,5,6].

Therefore, educational programs directed towards the prevention of occupational disphonia have be recommended for the control of vocal alterations and improvement in the quality of life of professionals who frequently use their voice [7-10]. Objective and clinical tests are commonly used in evaluating the effectiveness of vocal health programs, such as acoustic and perceptive voice analyses, which are forms of analyzing and quantifying voice quality changes [8,10,11]. Nevertheless, objective evaluations do not show the individual’s point of view of his/her psycho-emotional, social and professional problems that may be related to the changes in health [11].

The majority of studies in the literature have evaluated vocal educational programs by means of objective measurement instruments with focus on vocal characteristics [8,10,11]. Few studies have evaluated the biopsychosocial quality of the voice of subjects after participating in educational programs [7,10] and these showed the evaluation of subjects’ self-perception only in a quantitative manner, by means of scores, and did not present explored analysis of the responses.

In the field of vocal health, instruments to verify the inter-relationship between vocal problems and quality of life have been tested, such as, the Voice-Related Quality of Life (V-RQOL) [12]. A Brazilian version of V-RQOL was developed by Gasparini and Behlau [13]. The V-RQOL has been used by various researchers in the area of Phonoaudiology to investigate the relationships between quality of life and voice in teachers and subjects with and without vocal alterations, in addition to being pointed out as an important instrument for evaluating the impact of dysphonia on subjects’ lives.

Analysis of the quality of life with regard to vocal health has been the focus of researches conducted in cross-sectional and clinical studies [2,3,5]. However there is a need for studies that evaluate the impact of vocal health programs that are collective in scope, with regard to the quality of life of subjects in a longitudinal study.

Evaluating the effectiveness of vocal health programs by instruments after an intervention may be considered an important factor in planning public health policies.

The aim of this study was to make a longitudinal evaluation of the impact of voice educational activities on the quality of teachers’ lives, by means of a Quality of life and voice questionnaire and analyzed the results in an exploratory manner.

## Methods

### Sample

The population of the present study was composed of teachers from the public school network in the municipality of Piracicaba, SP, Brazil. All public schools (66) were divided into administrative regions (5) and eleven schools were randomly selected take into consideration number of schools by region. The randomization process, using the school as the sampling unit, was chosen for two reasons: a) teachers had socioeconomical (socioeconomical status) and professional variables (workload in hours/week, number of years taught) very similar and did not differ significantly between schools (p > 0.05), b) teachers typically had a very high workload (most over 32 hours/week) and had only 2 hour meeting per week, in part, been met by the activities of the preventive program voice.

All teachers of the 11 schools selected were invited to participated and following inclusion criteria were used: participants should be non smokers, present no organic pathologies of the larynx previously diagnosed by a doctor, or report of complaining of persisting hoarseness for longer than 2 weeks, not doing speech therapy, not be over the age of 55 years. The age limit was established in order to prevent the characteristic of voice aging from being a study bias [14]. Seventy teachers which met the inclusion criteria signed a term of free and informed consent approved by the Research Ethics Committee of the Piracicaba School of Dentistry (Protocol nº 041/2009). The sample was composed by 30 subjects in the experimental group (26 women and 4 men with a mean age of 41.53 ± 7.01) and 40 in the control group (31 women and 9 men a mean age of 42.42 ± 7.71). The researchers decided *a priori* to random schools and allocation them into control and experimental group. The teachers from each school of the control and experimental groups participated of the program in the school in which they worked.

For sample calculation of the control and experimental group, a minimum of degrees of freedom was considered for the residue of the analysis of variance, with the minimum estimated size for each group being 13, which provided a test power of 0.8 for the level of significance of 0.05.

### Questionnaire

All participants were required to complete a short demographic questionnaire at the beginning of the study to enable the researches to gain information about signs and symptoms of dysphonia and the vocal use patterns at work. The answers to the questions in the questionnaire were closed and varied within a Likert scale, which corresponded to the categories: never, rarely, sometimes, always, do not know. Responses were dichotomized between yes (sometimes and always) and not (never, rarely and do not know).

#### Procedures

The subjects in the control group participated in 2 lectures covering guidance on vocal hygiene habits. For the participants in the experimental group 1 lecture was held on vocal hygiene habits and 4 meeting with training exercises specifically for the voice. The guidance sessions had a 30-minute duration. The meetings were held at 15-day intervals.

#### Educational activities: vocal hygiene

Initially, the participants were informed about how the voice is produced, and which are the pathologies that may affect the vocal tract, harming the voice. Afterwards, the subject were instructed as regards the practice of healthy voice habits.

The scope of vocal hygiene habits focuses on the importance of drinking water during professional activity, and the beneficial effect of eating apples on the vocal tact, acting as astringent of the vocal tract mucosa, and vocal rest as an action that must be practiced in the interval after work [11,15,16]. Participants were instructed to avoid habits that are harmful to the voice, such as: speaking loudly, shouting, throats clearing, speaking over background noise, speaking loudly, speaking overloads in uncomfortable positions such as speaking while leaning down, the use of sprays and lozenges, constant ingestion of cold drinks, as well as exposing the body to abrupt temperature changes without self-protection by wearing a coat or suitable clothing for the situation, practicing physical activities or other activities including vocal use and poor sleep quality [15,16].

The speech therapist discussed information with the participants, on strategies to obtain students attention in the classroom. Among the strategies indicated were replacing the habit of shouting by other means, such as clapping or blowing a whistle to draw students attention in the classroom. In addition, they were instructed about the importance of facing the student when giving explanatory lessons, avoiding speaking while writing on the blackboard, and reducing overload on the vocal tract, caused by tension in the cervical region [4].

At the beginning of each meeting, a discussion was held among the participants of the group, with the purpose of reflecting about the subject that would be approached. Multimedia resources were used to transmit the content of lectures. The participants received a folder containing explanatory matter on the subject and a plastic bottle to get used to the habit of drinking water while they were teaching.

#### Educational activities: training exercises

In the experimental group 4 voice training exercise sessions were applied. The sessions separately approached the following topics: a. posture and cervical relaxation, b. respiration, c. phonation, frequency and intensity, d. resonance and articulation. In each session, 15 minutes was devoted to a theoretical approach to the subject, and 15 minutes to training the exercise. Three series of exercises were performed with intervals of 30 seconds. Each series, with 10 repetitions, totaling 30 repetitions of each approach.

a. Body Posture and Cervical Relaxation Exercises

Correct body posture related to the vertical axis of the spine and head during work activities was taught, demonstrating pictures of various teachers in classrooms, an participants were asked which of the characters were in a posture without an overload of tension during the activity of teaching. After reflecting about adequate posture in the work environment, training exercises were performed for relaxation of the cervical region and larynx with the object of diminishing local tension and favor loosening up voice production. Cervical relaxation exercises involved a sequence of rotating the shoulders backwards and forwards, movement of head flexion and extension, rotating the head to the left and right and vice versa. Subjects were instructed to make rotary self-massaging movements in the region of the larynx, accompanied by descending movements sliding down the vertical axis of the neck [7].

b. Breathing Exercises

It was explained to the participants that voice emission demands the coordination of various muscles, particularly between the respiratory muscles themselves and the diaphragm. So that for voice production without tension and with control of speech intonation, balanced breathing is fundamental. The participants were instructed step by step to breathe moving the diaphragm and abdominal region, feeling the entry and output of air from this region. To increase the air flow, individuals were asked to inspire normally, hold their breath for 5 seconds, and then slowly breathe out the air through their mouths. This same procedure was performed while emitting the fricative phoneme/s/continuously and feeling the region of movement of the diaphragm [7,10].

c. Phonation, Frequency and Intensity Exercises

With the aim of improving the vibration and amplitude of the vocal folds, favoring a balanced vocal production, it was proposed that participants make vibrant sounds of the tongue in the tone of habitual frequency of speech, in ascending and descending scales, and at both weak and strong intensity. Vibrant sound exercises of the tongue and/or lips allow greater flexibility of the vocal chords, increase the wave like movement of the mucosa and sound projection without effort and tension [7,10,17].

d. Resonance and Articulation Exercises

The work of resonance was performed to favor the adequate use of some of the bone and supra-glottal cavities, such as the larynx and facial sinuses. The object of articulation was to favor an improvement in Articulatory precision of the words in speech and good harmony in vocal production. Sequences of resonance exercises with nasal sounds (/m/, /n/ and /nh/) were applied, involving association with all the vowels. The teachers were encouraged to feel the sensation of the paranasal resonators when emitting the sound, and were instructed to practice these exercises in the morning, in order to improve the balance of the voice resonators. Emission of the humming sound was also practiced with the same objective as that of achieving equilibrium of resonance. To improve Articulatory precision, the emission of each consonant with each vowel was requested, exploring the articulatory point projected, and ample masticatory movements associated with the nasal sound [7,10,18].

The teachers were instructed to practice the vocal exercises and healthy habits on a day-to-day basis, and to do so, were instructed by means of a weekly time schedule containing the quantity and frequency of activities to be practiced.

A partnership was established with the schools with the fixation of murals in the access to teachers’ meeting rooms, containing instructions as regards the practice of the program activities in professional routine. In addition, in schools where there were no water filters in a place easily accessible to the teachers, the speech therapist spoke to the coordinators about providing a receptacle for this purpose.

#### Quality of life and voice evaluation

The Brazilian version (adaptation and translation) of the Voice-Related Quality of Life instrument (V-RQOL- Hogikyan and Seturaman) was applied in both groups at baseline and three months after conclusion of the educational program. This instrument has the capacity to evaluate the perception of subjects with regard to the impact of voice on their quality of life and may be used to follow-up the development in the clinical area and in planning vocal health promotion actions [5].

V-RQOL involves 10 questions, to which quality of life and voice are related, involving the Physical (Questions 1, 2,3,6,7 and 9), Socio-emotional (4,5,8 and 10) and Global (questions from 1 to 10) domains. For each response, judgment on a Likert scale is used, ranging between the least severity to the greatest severity of the problem. The scale corresponds to:1 = never happens and it is not a problem; 2 = hardly happens and rarely is a problem; 3 = sometimes happens and is a moderate problem; 4 = often happens and almost always is a problem; 5 = it always happens and really is a serious problem.

To calculate the final score of theV-RQOL, the rules generally applied in the majority of quality of life instruments were used. The standard score is calculated from the gross score, with a higher value indicating greater correlation between the voice and quality of life. The maximum score is 100 (best quality of life) and the minimum score is zero, for both the physical and socio-emotional domains, as well as the global domain.To calculate the scores, the following formula is used, a standard algorithm of this type of questionnaire:

$$\begin{array}{l} \;\frac{\text{Total}\;\text{Score}:100-\left(\text{total}\;\text{score}-10\right)\times 100}{40}\\ \;\frac{\text{Physical}\;\text{Functioning}\;\text{Score}:100-\left(\text{physical}\;\text{score}-6\right)\times 100}{24}\\ \frac{\text{Socio-emotional}\;\text{Score}:100-\left(\text{socio-emotional}\;\text{score}-4\right)\times 100}{16} \end{array}$$

#### Statistical analysis

After exploratory analysis of the data and selecting the best structure of covariance, the V-RQOL (total, emotional and physical) score data were analyzed by the methodology of mixed models for repeated measures (PROC MIXED), considering the level of significance of α < 0.05. Chi Square and Fischer tests were applied considering the level of significance of α < 0.05 to analyze responses of questionnaire.

For statistical analysis was used *SAS* 2008 version 9.1 software.

## Results

Table 1 showed that a high percentage of teachers complained of signs and symptoms for dysphonia such as hoarseness in the last 6 months (51.42%), vocal fatigue (60%), frequent clearing of the throat (52.85%), deep voice 37,14% and weak voice (38.57%). Regarding the use of voice refers to the act of teaching, 68.57% used the voice of an intense and continuous, and has the habit of shouting in the classroom.

**Table 1:** Frequency of responses regarding the presence of signs and symptoms for dysphonia and features the use of voice at work by teachers in the sample (n = 70)

**Questions about Voice**	**Yes n = 70**	**Total (%)**
**Signs and symptoms of vocal**		
Hoarseness in the last 6 months	36	51.42
Temporary loss of the voice	16	22.85
Breathlessness during speech	24	34.28
Realize that his/her voice is thin	9	12.85
Realize that his/her voice is thick	26	37.14
Realize that his/her voice is weak	27	38.57
Feeling the need to clear my throat	37	52.85
Feeling sore throat	29	41.42
Feeling tired when speaking	42	60.00
Feel your throat is dry	50	71.42
**Characteristics of Use**		
Voice use intensely	48	68.57
Yelling too much	48	68.00

Table 2 shows the descriptive and percentage analysis of the answers to the questions of the V-RQOL instrument. There are no significance inter groups (control and experimental) for all questions. However, in Table 3, for all the V-RQOL scores there were statistically significant difference in the comparison between the initial and final evaluation for control and experimental groups, but no statistical difference inter groups.

**Table 2:** Comparison of the answers pre and post educational intervention in the control e experimental groups of the V-RQOL

		**Control (n = 40)**				**Experimental (n = 30)**				**p-value**	**p-value**
**Questions**	**Categories**	**Pre**		**Post**		**Pre**		**Post**		**Pre**	**Post**
		**N**	**%**	**N**	**%**	**N**	**%**	**N**	**%**		
1. I have trouble speaking loudly or being heard in noisy situations.	never	10	25.0	15	37.5	4	13.3	7	23.3	0.1221	0.4704
	hardly	13	32.5	11	27.5	7	23.3	10	33.3		
	Sometimes	12	30.0	9	22.5	8	26.7	7	23.3		
	Often	4	10.0	5	12.5	5	16.7	4	13.3		
	always	1	2.5	0	0.0	6	20.0	2	6.7		
2. I run out of air and need to take frequent breaths when talking.	never	20	50.0	19	47.5	11	36.7	11	36.7	0.2415	0.4703
	hardly	7	17.5	11	27.5	8	26.7	7	23.3		
	Sometimes	11	27.5	8	20.0	5	16.7	7	23.3		
	Often	1	2.5	2	5.0	3	10.0	3	10.0		
	always	1	2.5	0	0.0	3	10.0	2	6.7		
3. I sometimes do not know what Will come out when I begin speaking.	never	15	37.5	22	55.0	16	53.3	17	56.7	0.5201	0.8061
	hardly	19	47.5	11	27.5	10	33.3	9	30.0		
	Sometimes	3	7.5	5	12.5	3	10.0	4	13.3		
	Often	2	5.0	2	5.0	0	0.0	0	0.0		
	always	1	2.5	0	0.0	1	3.3	0	0.0		
4. I am sometimes anxious or frustrated (because of my voice).	never	22	55.0	32	80.0	16	53.3	0	66.7	0.3061	0.4347
	hardly	12	30.0	5	12.5	5	16.7	6	20.0		
	Sometimes	6	15.0	3	7.5	7	23.3	4	13.3		
	Often	0	0.0	0	0.0	1	3.3	0	0.0		
	always	0	0.0	0	0.0	1	3.3	0	0.0		
5. I sometimes get depressed (because of my voice)	never	33	82.5	38	95.0	21	70.0	25	83.8	0.3279	0.1298
	hardly	6	15.0	2	5.0	5	16.7	5	16.7		
	Sometimes	1	2.5	0	0.0	3	10.0	0	0.0		
	Often	0	0.0	0	0.0	0	0.0	0	0.0		
	always	0	0.0	0	0.0	1	3.3	0	0.0		
6. I have trouble using the telephone (because of my voice)	never	29	72.5	34	85.0	23	76.7	24	80.0	0.4928	0.7800
	hardly	9	22.5	4	10.0	4	13.3	5	16.7		
	Sometimes	2	5.0	2	5.0	3	10.0	1	3.3		
	Often	0	0.0	0	0.0	0	0.0	0	0.0		
	always	0	0.0	0	0.0	0	0.0	0	0.0		
7. I have trouble doing my job or practicing my profession (because of my voice).	never	24	60.0	30	75.0	14	46.7	20	66.7	0.2502	0.6943
	hardly	11	27.5	5	12.5	6	20.0	6	20.0		
	Sometimes	2	5.0	4	10.0	4	13.3	3	10.0		
	Often	3	7.5	0.0	0.0	4	13.3	1	3.3		
	always	0	0.0	1	2.5	2	6.7	0	0.0		
8. I avoid going out socially (because of my voice).	never	36	90.0	39	97.0	29	96.7	29	96.7	0.7869	1.000
	hardly	3	7.5	1	2.5	1	3.3	1	3.3		
	Sometimes	1	2.5	0	0.0	0	0.0	0	0.0		
	Often	0	0.0	0	0.0	0	0.0	0	0.0		
	always	0	0.0	0	0.0	0	0.0	0	0.0		
9. I have to repeat myself to be understood.	never	19	47.5	23	57.5	11	36.7	17	56.7	0.8407	0.6138
	hardly	14	35.0	12	30.0	14	46.7	12	40.0		
	Sometimes	5	12.5	4	10.0	3	10.0	1	3.3		
	Often	1	2.5	0	0.0	1	3.3	0	0.0		
	always	1	2.5	1	2.5	1	3.3	0	0.0		
10. I have become less outgoing (because of my voice).	never	31	77.5	37	92.5	24	80.0	30	100.0	0.5478	1.000
	hardly	7	17.5	1	2.5	3	10.0	0	0		
	Sometimes	1	2.5	1	2.5	2	6.7	0	0		
	Often	1	2.5	1	2.5	0	0.0	0	0.0		
	always	0	0.0	0	0.0	0	0.0	0	0.0		

There was no statistically significant difference inter groups (control and experimental) (p > 0.05). Chi Square and Fisher tests.

**Table 3:** Comparison of the mean (MD) and standard deviation (SD) of overall scores, emotional and physical of V-RQOL, in the control group (n = 40) and experimental (n = 30) groups, pre and 3 months post the voice educational program

**Overall the escores of the V-RQOL**	**Groups**	**Evaluations**			
		**Pre**		**Post**	
		**MD**	**SD**	**MD**	**SD**
Total Escore	Control	84.9 *	11.2	89.4	10.8
	Experimental	80.9 *	17.2	87.1	11.4
Social-Emocional Escore	Control	92.3 *	9.2	96.9	6.9
	Experimental	89.0 *	17.0	95.8	6.4
Physical Functioning Score	Control	82.0 *	13.1	85.9	13.4
	Experimental	75.6 *	19.4	83.2	14.6

* Differs from the post-evaluation (p < 0.05). Test Proc MIXED.

## Discussion

In the present study, the mean scores ranged between 75.6 and 92.5 in the control and experimental groups in the pre- educational program situation. In the study conducted by Spina et al. [19], when the quality of life and voice were correlated with levels of dysphonia and professional activity, scores from 71 to 100 points of the V-RQOL were found for individuals with better quality of life and from 0 to 35 points for the group with worse quality. In the V-RQOL validation study, used for dysphonic individuals, means of 53.5 for the total score, 55.9 for the socio-emotional domain and 51.9 for the physical domain were found, whereas for individuals with a normal voice all the scores were over 70 [12].

The mean scores of the present study suggest that the quality of life of the subjects was not being interfered with by dysphonia, since the V-RQOL scores were found to be relatively high and close to 100. Although subjects of this sample, both the control and experimental, have reported signs and symptoms for dysphonia, they do not associate these symptoms as negatively impacting on quality of life. The results corroborate the findings of Grillo and Penteado (2005) who studied the impact of voice on the quality of life of primary school teachers. This leads one to reflect on the need for self-perception of teachers as regards use of the voice in day-to-day routine, as well as the impact that vocal alterations and health problems may have on their quality of life.

Possibly there is greater need for these professionals to identify their respective voice problems, which may interfere in their day-to-day activities. Although the focus of the educational program did not contemplate training for auditory self perception of the voice and vocal psychodynamics, these aspects may be suggested for application in future vocal health programs for teachers.

After the educational activities, teachers showed significantly higher domain and overall V-RQOL scores after preventive intervention, in both control and experimental groups, showing that these activities had a positive impact on the participants’ lives. This shows that both the activities provided with guidance on vocal hygiene, and those including practice of the exercises reflected positively on the quality of life of subjects.

It is important to point out the importance of educational actions on teachers’ vocal health, reflecting on the individuals’ quality of life. It is known that instructions such as taking care of hydration and perceptive measures such as, for example, not shouting in the classroom and not speaking with strong intensity in the presence of noise may improve the teacher’s vocal quality [4]. Hydration promotes and maintains healthy functioning of the larynx, especially in individuals that use the voice professionally [15]. On the other hand, dehydration may increase phonatory effort, contributing to the manifestation of vocal fatigue, particularly for professionals who use the voice as an instrument for work [20]. Instructions about the habit of drinking water during the professional routine were worked on in this study in both groups, by means of lectures, discussions on the subject among the participants, explanatory folders and a 30 ml bottle offered to each participant to use for drinking water during day-to-day work. Other educational measures were also transmitted, such as not shouting, but drawing the pupils attention by means of other resources such as clapping their hands or using a whistle. Emphasis on the practice of changing to healthy behaviors for the voice in both groups positively favored the quality of life of participants, observed in the global dominion score of the V-RQOL.

The present study differs from other educational programs in which they verified improvements in the quality of life of the participants in educational programs, but only evaluated in situations of vocal training exercises [7,15,21]. The fact that 2 lecture sessions were developed on vocal hygiene habits in the control group, in addition to the resource of offering a botttle of water, differs from the methodology of other studies. These studies approached the subject of vocal hygiene habits in a single session only for the control group [7,9,10].

Various authors have mentioned the biopsychosocial impact in the face of voice problems that affect teachers [6,21]. Studies have shown that when evaluating the impact of educational programs for voice professionals by means of protocols with measurement of qualitative and quantitative measures, it was possible to observe a significant improvement with regard to physical and emotional aspects in general [7,21]. These effects were better observed in intervention programs related to voice training exercises associated with vocal hygiene habits [7,9,22]. In the present study there was improvement in the aspects of vocal health, intensifying the improvement of physical and psychic well being both in the control and experimental groups.

Studies have shown that educational actions of a preventive nature, when developed in groups and in the work environment may improve the quality of life of workers, particularly in physical and psychic well being [4,22-24]. Researchers have indicated that the fact of an individual participating in group educational activities with persons who have similar problems and difficulties favors improvement in psychic well being, providing a reduction in stress and anxiety at work and improvement in communication [25]. A hypothesis for the findings of reduction in anxiety and frustration of individuals faced with voice difficulties, observed in the present study, may be that the dynamics of discussing the subjects raised in groups, provided a support network among the teachers. Timmermans et al. (2004) [26] observed significant chance in the emotional aspects of voice professionals who participated in an educational program with instructions about vocal hygiene and in situations of vocal training exercises, in addition to verifying an improvement after 18 months with regard to psycho-emotional aspects, both in the group given vocal hygiene instructions and the group with training exercises, concluding that this improvement, for both groups, reflected maturation as regards self perception and better control of feelings over the course of time.

In the present study, statistically significant difference was observed for the physical score of the V-RQOL for both the control and experimental group. The findings differ from those of the study of Duan et al. (2010), who evaluated the quality of life of subjects who participated in a vocal health program. There was a report of improvement in the physical and functional aspects of the voice only in the experimental group. These authors provided a lecture on vocal hygiene for the control and experimental groups, in addition to 4 sessions of training exercises for the latter group. Although the number of intervention sessions applied to the control and experimental group in the study of Duan et al. (2010) are compatible with those of the present study, the findings for the control and experimental groups obtained statistically significant results in the final evaluation of the physical score. It is suggested that in the present study, the physical improvement reported by the control group is due to the emphasis on healthy practices for the voice, reinforced in two lectures. The fact of instructing teachers about drinking water during the time they are giving lessons may result in beneficial effects when they are incorporated by the subjects, due to the reduction in friction between the vocal folds and in the reduction of the effort to speak. The same educational practices were discussed in the experimental group, and the teachers were encouraged to practice them together with the training exercises.It is suggested that the instructions transmitted were assimilated in good part by the teachers in both groups, which may result in positive effects on the improvement of the physical and functional aspects of the voice.

In general terms, although we found no difference intra and inter groups for each question of V-RQOL, we see a pattern of change to higher percentages for the categories never and hardly to the two groups, suggesting educations actions, can improve the quality of life of the subjects in relation to biopsychosocial aspects, such as improvement in psychological aspects, in communication and in the activities related to work.

One of the interesting points of this study was the randomization process. Initially, schools were randomly selected and then a general questionnaire, concerning socioeconomic and professional information, was applied to all teachers. Based on the results, we found no differences between the characteristics of teachers in different schools. Thus, there was the final draw of the schools, divided into experimental and control groups. This option was due to the fact that teachers have their workload too long (most with more than 32 hours/week) and they have only 2 hours/week available for meetings, part of this time in which the activities were carried out the study. In practical terms, it would be almost impossible to divide the sample into two study groups for ethical and logistical reasons. This form of randomization was similar to the study of Pasa et al. (2007), randomization of the sample of schools to compose groups, and different of other studieswhich the total sample of selected individuals was randomized between control and experimental group [7,8,21].

A limitation of this study is related to the number of male included in the experimental group (n = 7), and this is explained by low number of male teaching in public schools (less than 15% of the total) and low level of adhesion by male in the selected schools. However, due the fact men are less exposed to vocal problems than women due to the larynx and vocal folds conformation, this could exert a little impact on results. Another potential limitation was the limited number of vocal exercise sessions (4), possibly being a bias to identify gains in the voice quality longitudinal. The sessions of vocal health program was only possible to be realized in the form of separate schools, it was possible to gather all who were part of the same group at a single time and place.

Thus, it is important for future vocal health programs for teachers envisage the inclusion of both educational activities with vocal hygiene instructions and specific training exercises to obtain and improvement in the quality of life of subjects. This shows that there is a need for partnership between the public health area and the educational area, so that inter-sectorial actions promote quality of life at work, specifically for teachers.

## Conclusion

The vocal health educational actions had a positive effect on the quality of life and voice of teachers both from the psycho-emotional aspects, and on improvement in the functional aspects of the voice.

## Abbreviations

V- RQOL: Voice: related quality of life; MD: Mean; SD: Standard deviation; Hz: Hertz; dB: Decibel.

## Competing interests

The authors declare that they have no competing interest.

## Authors’ contributions

RAP participated in the conception and design of the study, data interpretation, data acquisition, and drafting the manuscript. MIBCR contributed to the conception and design and revision critical of the study. MCM contributed to critical revision of manuscript. GMBA participated in data analyses. FLM participed in the conception and design of the study and critical revision. ACP participed in the conception and design of the study and critical revision. All authors read and approved the final manuscript.
